# Trends in reperfusion treatments, functional outcomes and mortality for first-ever ischaemic stroke in Norway from 2014 to 2021: The Norwegian Stroke Registry

**DOI:** 10.1177/23969873251331482

**Published:** 2025-04-13

**Authors:** Kevin C Elangwe, Ellisiv B Mathiesen, Torunn Varmdal, Bent Indredavik, Agnethe Eltoft

**Affiliations:** 1 Department of Clinical Medicine, UiT the Arctic University of Norway, Tromsø, Norway; 2 Department of Neurology, University Hospital of North Norway, Tromsø, Norway; 3 Department of Circulation and Diagnostic Imaging, NTNU, Trondheim, Norway; 4 Department of Medical Quality Registries, St. Olav’s University Hospital, Trondheim, Norway; 5 Department of Neuromedicine and Movement Science, Faculty of Medicine and Health Sciences, NTNU, Trondheim, Norway

**Keywords:** Time trends, reperfusion therapy, functional outcome, mortality, nationwide registry, ischaemic stroke

## Abstract

**Introduction:**

Acute ischaemic stroke (AIS) treatment has undergone major changes in the last decades with regards to reperfusion treatment with intravenous thrombolysis (IVT) and mechanical thrombectomy (MT). We analysed temporal trends in reperfusion treatment, functional outcomes and mortality among patients with first-ever AIS.

**Patients and Methods:**

We included 45,686 first-ever AIS patients registered in the Norwegian Stroke Registry from 2014 to 2021. Temporal trends in reperfusion therapy, functional outcome defined by modified Rankin Scale (mRS) score at 90 days and mortality were assessed in age-and sex-adjusted logistic regression models and in analyses stratified by age and reperfusion treatment.

**Results:**

Mean age was 73.8 years and 54.5% were men. The use of reperfusion treatment increased over time (IVT only from 15.5% to 18.1%; MT only from 0.4% to 2.8%; IVT + MT combined, from 0.9% to 3.4%). The proportion of patients achieving mRS 0–2 at 90 days increased from 64.2% to 68.1%. The 90-day mortality decreased from 11.7% to 10.5%. Improvement in 90-day functional outcome was most notable in patients receiving IVT, but was also observed in patients not receiving reperfusion treatment. Patients aged ⩾80 years showed improvement in functional outcome and reduced mortality rate, while less noticeable time trends were observed in patients <80 years.

**Discussion and conclusion:**

Reperfusion therapy for first-ever AIS increased significantly over time, concurrent with significant improvements in functional outcome and lower mortality rate. Improvements in outcome were more prominent in the older population. Improved outcome among non-reperfused patients suggest that factors other than reperfusion therapy contribute to these results.

## Graphical abstract

**Graphical abstract:**
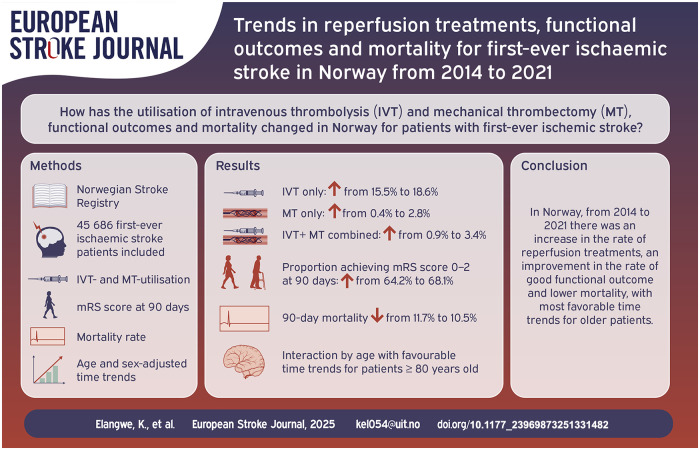

## Introduction

Randomised clinical trials demonstrate that reperfusion therapies, specifically intravenous thrombolysis (IVT) and mechanical thrombectomy (MT) significantly improve functional outcome in patients admitted with acute ischaemic stroke (AIS).^1,2^ Follow-up trials have expanded the eligibility criteria for reperfusion treatment, including older age and extended treatment window.^3–6^ The efficacy of reperfusion treatment is highly time-dependent,^7,8^ with pre- and post-admission delays reducing accessibility and effectiveness, timely implementation remains a challenge in many countries and healthcare systems.^9,10^

Despite advances in AIS treatment, recent nationwide studies on IVT and MT utilisation, functional outcomes, and mortality among European AIS patients are limited. American cohort studies report increased reperfusion treatments over time and decreased mortality in AIS patients.^11,12^ European studies report increased reperfusion rates,^13–15^ but while improved outcomes were noted in the early 2000s,^13,14^ updated data on trends in functional outcomes and mortality are scarce. This study aims to describe trends in reperfusion treatments, functional outcomes and mortality for first-ever AIS-patients in the Norwegian Stroke Registry (2014-2021) and assess variations by sex, age, stroke severity, and hospital size.

## Patients and methods

### Norwegian Stroke Registry (NSR)

The NSR is a nationwide medical quality registry, first established in 2012. Norwegian law requires hospitals to report all adult stroke hospitalisations (⩾18 years) admitted within 28 days of symptom onset, diagnosed with International Classification of Diseases and Related Health Problems 10th Revision (ICD-10) codes I61 Intracerebral haemorrhage, I63 Cerebral infarction or I64 Stroke, unspecified. Physicians or trained nurses complete a web-based form upon discharge, and patient reported outcomes after 90 days follow-up is obtained through self-administered questionnaires from the patient or caregiver, via telephone interview or at outpatient consultations. Mortality data are updated by cross linkage between NSR and the Norwegian Cause of Death Registry.

## Inclusion and exclusion criteria

The study included all first-ever AIS patients (ICD-10 code I63) hospitalised between January 1, 2014, and December 31, 2021. In total, 59,860 AIS cases were registered in the study period. We excluded cases with previous stroke (*n* = 13,328) or missing data regarding previous stroke (*n* = 846) resulting in 45,686 first-ever AIS patients included in the study.

## Patient characteristics, treatments and outcomes

Patient data included information on age, sex, living conditions, current smoking, atrial fibrillation (AF), diabetes mellitus, myocardial infarction (MI), transient ischaemic attack (TIA), use of medications at admission (antihypertensive, lipid-lowering, oral anticoagulant and platelet inhibitors) and pre-stroke modified Rankin Scale (mRS) score. Stroke severity was reported as National Institutes of Health Stroke Scale (NIHSS) score (0–42) divided into <5, 5–15 and >15 subgroups. Treatment variables included treatment with IVT, MT and stroke unit care (all yes/no). Hospital size was divided in three groups according to annually admitted stroke patients (0–99, 100–299 and ⩾300 hospitalisations). Time from symptom onset to IVT was categorised as <3, 3–4.5 and >4.5 h. Time from symptom onset to MT was categorised as <6 and ⩾6 h. Wake-up strokes and other strokes of unknown symptom onset were treated as a separate category. Functional outcome was assessed by 90-day mRS-score, categorised into excellent (0–1), good (0–2), moderate (3–4) and poor (5–6) subgroups. Complete mortality data at 30 days, 90 days and 1 year was obtained through linkage between the NSR and the Norwegian Cause of Death Registry.

## Statistical analyses

Analyses were conducted using RStudio 4.3.2. Continuous variables are presented as means with standard deviations (SD), and categorical variables as relative frequencies. Yearly frequency tables display time trends in reperfusion treatment, stroke unit utilisation, time metrics and outcomes (mRS score, discharge home, and 30-day, 90-day and 1-year mortality). Treatment options were divided in mutually exclusive categories as IVT alone, MT alone, IVT + MT and No IVT/MT. Binary logistic regression analyses, adjusted for age and sex, assessed time trends for each treatment option and functional outcome for the overall population and for each treatment group seperately. Interactions by age, sex, baseline NIHSS-score and hospital size were evaluated using cross-products of these variables with time in adjusted models. A two-sided *p*-value < 0.05 indicated statistical significance. Trend analyses were complete case analysis, with model selection based on Bayesian Information Criteria for first- and second-degree logistic regression models.

## Results

A total of 45,686 patients were treated for first-ever AIS from 2014 to 2021. Baseline characteristics are presented in Table 1. The mean age was 73.8 years (SD 13.5) and the proportion males was 54.4% in the AIS population. On average, 93.3% of the population lived at home prior to AIS occurrence and 89.5% had a good prestroke mRS-score (0–2). The proportion of current smokers decreased over time (26.1%–21.9%), similar changes were observed for previous TIA (9.6%–6.9%), AF (25.5%–22.8%) and MI (13.4%–10.6%). The proportion of patients using preventive antihypertensives, statins and oral anticoagulants increased over time, whereas use of platelets inhibitors decreased (34.9%–26.5%). The proportion of patients presenting with mild symptoms (NIHSS score <5) increased from 61.8% to 66.6%.

**Table 1.** Baseline characteristics of first-ever ischaemic stroke patients, stratified by year.

Baseline characteristics	2014 *n* = 5358	2015 *n* = 5443	2016 *n* = 5583	2017 *n* = 5674	2018 *n* = 5763	2019 *n* = 5914	2020 *n* = 5927	2021 *n* = 6024
Mean age, years (SD)	73.6 (13.7)	73.9 (13.5)	74.0 (13.8)	73.7 (13.4)	73.9 (13.5)	73.6 (13.5)	73.3 (13.3)	74.0 (13.1)
Men, *n* (%)	2867 (53.5)	2913 (53.5)	3016 (54.0)	3086 (54.4)	3148 (54.6)	3199 (54.1)	3240 (54.7)	3292 (54.6)
Current smoking, *n* (%)	1181 (26.1)	1212 (26.9)	1160 (24.8)	1257 (25.7)	1144 (23.1)	1153 (23.4)	1167 (23.3)	1110 (21.9)
Prior myocardial infarction, *n* (%)	715 (13.4)	775 (14.3)	732 (13.2)	709 (12.5)	780 (13.6)	709 (12.1)	700 (11.9)	636 (10.6)
Prior TIA, *n* (%)	504 (9.6)	460 (8.6)	510 (9.2)	491 (8.7)	497 (8.7)	471 (8.0)	398 (6.8)	412 (6.9)
Diabetes, *n* (%)	932 (17.5)	918 (17.0)	902 (16.2)	970 (17.2)	1050 (18.3)	1095 (18.5)	1056 (17.9)	1062 (17.7)
Atrial fibrillation, *n* (%)	1349 (25.5)	1318 (24.5)	1385 (25.0)	1304 (23.1)	1366 (23.8)	1415 (24.0)	1351 (22.9)	1368 (22.8)
Use of medication, *n* (%)								
Antihypertensive drugs	2290 (43.6)	2384 (44.3)	3036 (54.8)	3084 (54.7)	3190 (55.7)	3218 (54.7)	3150 (53.5)	3212 (53.5)
Cholesterol-lowering drugs	1527 (28.8)	1499 (27.7)	1565 (28.2)	1618 (28.7)	1737 (30.3)	1802 (30.7)	1808 (30.7)	1855 (30.9)
Oral anticoagulants	516 (9.7)	534 (9.9)	596 (10.7)	656 (11.6)	725 (12.6)	743 (12.6)	786 (13.3)	806 (13.4)
Platelet inhibitors	1852 (34.9)	1826 (33.8)	1896 (34.2)	1788 (31.7)	1822 (31.8)	1791 (30.4)	1692 (28.7)	1593 (26.5)
Living at home, *n* (%)	4928 (92.9)	5017 (92.7)	5154 (92.9)	5235 (92.9)	5371 (93.7)	5511 (93.5)	5522 (93.5)	5660 (94.1)
Pre-stroke mRS, *n* (%)								
0	3529 (65.9)	3562 (65.5)	3287 (64.8)	3156 (62.4)	3326 (63.3)	3149 (59.7)	3204 (59.8)	3374 (59.6)
1	837 (15.6)	850 (15.6)	750 (14.8)	828 (16.4)	801 (15.2)	941 (17.8)	999 (18.7)	1062 (18.8)
2	460 (8.6)	483 (8.9)	485 (9.6)	523 (10.3)	576 (11.0)	605 (11.5)	624 (11.7)	624 (11.7)
3	346 (6.5)	339 (6.2)	365 (7.2)	374 (7.4)	366 (7.0)	394 (7.5)	357 (6.7)	454 (8.0)
4	167 (3.1)	183 (3.4)	171 (3.4)	156 (3.1)	165 (3.1)	163 (3.1)	146 (2.7)	134 (2.4)
5	19 (0.4)	25 (0.5)	18 (0.4)	18 (0.4)	23 (0.4)	22 (0.4)	24 (0.5)	10 (0.2)
NIHSS on admission, *n* (%)								
<5	2575 (61.8)	2550 (61.3)	2721 (64.1)	2812 (63.1)	3069 (64.9)	3143 (64.0)	3304 (66.7)	3385 (66.6)
5–15	1173 (28.2)	1184 (28.5)	1118 (26.3)	1200 (26.9)	1248 (26.4)	1337 (27.2)	1231 (24.8)	1260 (24.8)
>15	416 (10.0)	427 (10.3)	408 (9.6)	441 (9.9)	410 (8.7)	429 (8.7)	421 (8.5)	435 (8.6)

mRS: modified Rankin Scale; NIHSS: National Institutes of Health Stroke Scale.

The utilisation of IVT, MT and IVT + MT increased significantly during the study period (Table 2). Overall, IVT and MT utilisation among first-ever AIS increased (IVT 16.4% to 21.5%; MT 1.3%–6.2%). The percentage of patients treated with only IVT rose from 15.5% to 18.1%, only MT from 0.4% to 2.8% and IVT + MT combined from 0.9% to 3.4%. An increasing trend was seen in the following time windows for IVT (<3, 3–4.5 h and wake-up/unknown symptom onset) and MT (<6, ⩾6 h and wake-up/unknown symptom onset). No increase was seen for IVT in the >4.5 h time window.

**Table 2.** Time trends in treatment and time aspects, stratified by year.

Treatment and time aspects	2014	2015	2016	2017	2018	2019	2020	2021	*p*-Value*
IVT, *n* (%)	828 (15.5)	886 (16.3)	971 (17.4)	1098 (19.4)	1110 (19.3)	1102 (18.7)	1100 (18.6)	1091 (18.1)	<0.0001
MT, *n* (%)	22 (0.4)	30 (0.6)	46 (0.9)	55 (1.0)	119 (2.1)	174 (3.0)	155 (2.6)	170 (2.8)	<0.0001
IVT + MT, *n* (%)	47 (0.9)	74 (1.4)	98 (1.8)	159 (2.8)	188 (3.3)	221 (3.8)	186 (3.1)	204 (3.4)	<0.0001
No IVT/MT, *n* (%)	4442 (83.2)	4446 (81.8)	4433 (79.9)	4346 (76.8)	4329 (75.3)	4393 (74.6)	4461 (75.4)	4539 (75.5)	<0.0001
Stroke unit, *n* (%)	4940 (92.2)	5107 (93.8)	5184 (92.9)	5307 (93.5)	5381 (93.4)	5663 (95.2)	5661 (95.5)	5800 (96.3)	<0.0001
Time from symptom-onset to thrombolysis, *n* (%)									
<3 h	656 (12.2)	730 (13.4)	767 (13.7)	928 (16.4)	934 (16.2)	918 (15.5)	881 (14.9)	899 (14.9)	<0.0001
3–4.5 h	131 (2.4)	147 (2.7)	187 (3.4)	167 (2.9)	182 (3.2)	201 (3.4)	209 (3.5)	171 (2.8)	0.018
⩾4.5 h	16 (0.3)	15 (0.3)	14 (0.3)	16 (0.3)	11 (0.2)	16 (0.3)	22 (0.4)	19 (0.3)	0.510
Wake-up and unknown-onset stroke	53 (1.0)	61 (1.1)	78 (1.4)	102 (1.8)	112 (1.9)	135 (2.3)	129 (2.2)	134 (2.2)	<0.0001
Time from symptom-onset to thrombectomy, *n* (%)									
<6 h	48 (0.9)	66 (1.3)	87 (1.6)	131 (2.3)	177 (3.1)	203 (3.5)	151 (2.6)	189 (3.1)	<0.0001
⩾6 h	4 (<0.1)	7 (0.1)	15 (0.3)	26 (0.5)	46 (0.8)	59 (1.0)	59 (1.0)	53 (0.9)	<0.0001
Wake-up and unknown-onset stroke	6 (0.1)	12 (0.2)	18 (0.3)	27 (0.5)	47 (0.8)	59 (1.0)	51 (0.9)	54 (0.9)	<0.0001

IVT: intravenous thrombolysis, MT: mechanical thrombectomy.

Percentages refer to proportion of all patients.

* *p* for time trend, assessed by binary logistic regression adjusted for age and sex.

Trends in functional outcome and mortality are shown in Table 3. Patients with a good mRS score at 90 days significantly increased from 64.2% to 68.1%, and discharge home rose from 50.3% to 53.8%. In contrast, a decline was seen in obtaining a poor mRS-score (18.0%–15.0%), whereas no time trend was observed for obtaining excellent and moderate mRS-score. The 30-day, 90-day and 1-year age and sex-adjusted mortality rates all significantly decreased (8.4% to 7.5%; 11.7% to 10.5%; 17.9% to 15.5%; respectively).

**Table 3.** Time trends in functional status, discharge destination and mortality at follow-up, stratified by year.

Outcome	2014	2015	2016	2017	2018	2019	2020	2021	*p*-Value*
Functional status at 90-day follow-up, *n* (%)									
Excellent (mRS 0–1)	1910 (49.4)	1915 (45.1)	1964 (46.4)	2100 (47.2)	2161 (47.0)	2159 (45.5)	2453 (49.4)	2427 (49.2)	0.154
Good (mRS 0–2)	2481 (64.2)	2714 (63.9)	2791 (65.9)	2911 (65.4)	3104 (67.5)	3179 (67.0)	3325 (66.9)	3361 (68.1)	<0.0001
Moderate (mRS 3–4)	689 (17.8)	791 (18.6)	765 (18.1)	785 (17.6)	759 (16.5)	788 (16.6)	887 (17.9)	831 (16.8)	0.114
Poor (mRS 5–6)	695 (18.0)	739 (17.4)	676 (16.0)	756 (17.0)	737 (16.0)	781 (16.4)	756 (15.2)	742 (15.0)	0.0027
Discharge home, *n* (%)	2691 (50.3)	2722 (50.2)	2821 (50.6)	2813 (49.6)	2927 (50.8)	2965 (50.1)	3145 (53.1)	3241 (53.8)	<0.0001
Mortality rate at different time points from stroke onset, *n* (%)									
30 days	448 (8.4)	475 (8.7)	442 (7.9)	493 (8.7)	444 (7.7)	439 (7.4)	468 (7.9)	450 (7.5)	0.036
90 days	624 (11.7)	652 (12.0)	586 (10.5)	661 (11.7)	626 (10.9)	632 (10.7)	617 (10.4)	631 (10.5)	0.019
1 year	956 (17.9)	948 (17.4)	917 (16.4)	1019 (18.0)	979 (17.0)	975 (16.5)	921 (15.5)	931 (15.5)	0.0003

mRS: modified Rankin Scale.

* *p* for time trend, assessed by binary logistic regression adjusted for age and sex.

**Figure 1.**
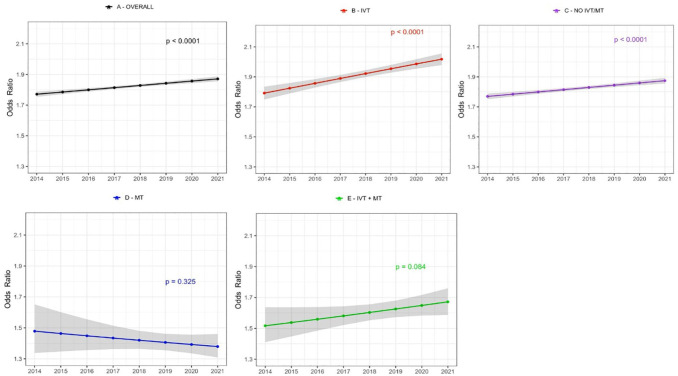
Time trends in age and sex adjusted odds ratio for achieving good functional outcome (mRS-score 0–2) at 90-day after first-ever acute ischaemic stroke stratified by treatment group. Shaded area represents 95% confidence intervals. (a) All patients, (b) IVT treated patients only, (c) patients not treated with IVT or MT, (d) MT treated patients only, (e) patients treated with both IVT + MT.

**Figure 2.**
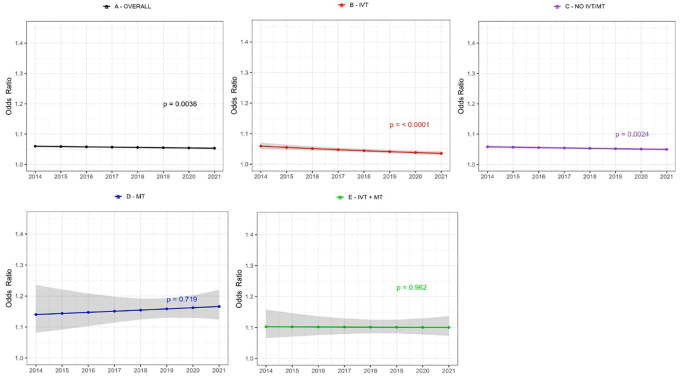
Time trends in age and sex adjusted odds ratios for 90-day mortality after first-ever acute ischaemic stroke stratified by treatment groups. The shaded area represents 95% confidence interval. (a) All patients, (b) IVT treated patients only, (c) patients not treated with IVT or MT, (d) MT treated patients only, (e) patients treated with both IVT + MT. IVT: intravenous thrombolysis, MT: mechanical thrombectomy.

Patients in the IVT - and No IVT/MT groups showed a significant increase in achieving good functional outcome (mRS 0–2) at 90-day follow-up, while no trend was observed in the MT or IVT + MT group. The IVT-group had the highest increase (54.8%–68.5%). There was a significant decline in 90-day mortality rate in the IVT- and No IVT/MT group. The greatest decline was in the IVT-group (11.9% to 9.0%), with the No IVT/MT-group decreasing from 11.6% to 10.2%; no significant trends were observed in the MT and IVT + MT groups (Supplemental Table 3 and Figures 1 and 2).

**Figure 3.**
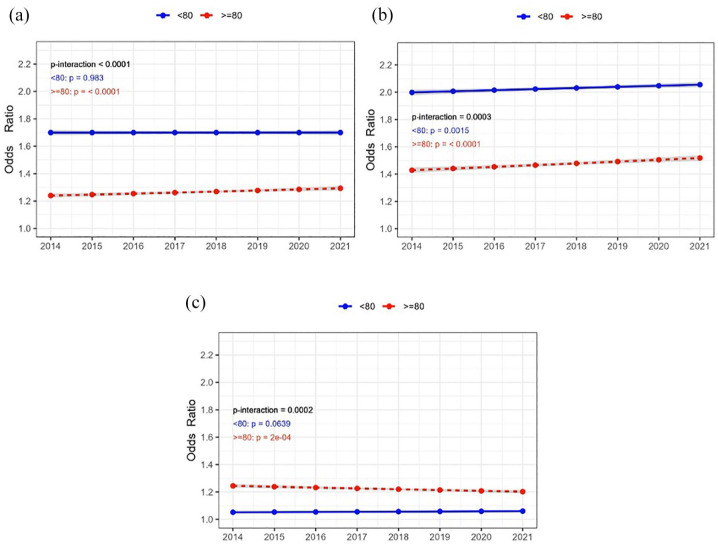
Time trends in sex-adjusted odds ratios for: (a) excellent functional outcome (mRS 0-), (b) good functional outcome (mRS 0–2) and (c) mortality at 90-days after first-ever acute ischaemic stroke. Results are shown for patients aged ⩾80 years (red lines) and <80 years (blue lines). The shaded area represents 95% confidence interval.

Significant interaction by age was observed for 90-day functional outcome. Increasing trends in achieving excellent (mRS 0–1) and good functional outcome (mRS 0–2) was most prominent to patients aged ⩾80 years (Figure 3, Supplemental Table 2, Supplemental Figures 1 and 2). Similarly, 30-day, 90-day and 1-year mortality decreased in the patients ⩾80 years, but remained stable in younger patients (Table 2, Supplemental Figure 1). Utilisation of MT and IVT + MT varied by hospital size, showing the highest utilisation and increase over time in larger hospitals (Supplemental Table 2). Further interactions and stratified analyses are displayed in Supplemental Table 2 and Supplemental Figures 1 and 2.

## Discussion

In this nationwide study, reperfusion treatment rates increased significantly over time among first-ever AIS-patients. This was accompanied by a trend towards better functional outcomes and reduced 30-day, 90-day and 1-year mortality rates. Improvement in functional outcome was most notable among IVT-treated patients but also observed in those not receiving reperfusion treatment. Patients aged ⩾80 years showed improvement in functional outcome and reduced mortality rate, while less prominent time trends were observed in patients <80 years.

The rise in reperfusion treatment rates aligns with other studies^11–15^ and occurred in a period with major advances within AIS care, particularly implementation of MT, which overall surged from 1.3% to 6.2% during the study period after its inclusion in national guidelines in 2017^16^ based on pivotal trials.^2,17^ Its peak in 2018 and 2019 likely reflects the extension of the treatment window to 24-h post-symptom onset, based on trials published in 2017–18.^5,6^ IVT-rates showed a similar trend, likely reflecting extension of treatment window and including wake-up patients and those over 80 years old as treatment candidates.^3,4,18,19^ Additionally, targeted public awareness campaigns and optimised emergency protocols may have contributed to increased IVT utilisation.^20,21^

Our findings demonstrate the rapid integration of trial evidence into clinical practice, as shown by increased reperfusion treatment in extended time windows. Norwegian reperfusion treatment rates for all AIS-patients peaked in 2019 (IVT 22%; MT 6.3%),^22^ consistent with our observations for first-ever AIS-patients. The plateauing of these trends towards the study’s end may be related to the COVID-19 disruptions. Norway maintains high accessibility to reperfusion treatments despite large and sparsely populated geographical areas, exceeding many European countries and aligning with the 2030 European Stroke Action Plan objectives.^23^ In 2021, 6.2% of the first-ever ischaemic stroke population received MT, while 8.6% had NIHSS score >15. This subgroup with NIHSS score >15 includes about half of all patients with large vessel occlusion,^24^ suggesting a potential for increased MT utilisation. Although further increases in MT rates may be challenging, variations in utilisation rates between small and larger centres highlight potential areas for improvement.

Improved functional outcomes in the overall population, including those not receiving IVT or MT, likely reflect advancements in stroke care such as increased stroke unit utilisation,^25^ as well as changes in the stroke population. Previous studies have suggested a shift towards a patient population with lower risk factor levels as well as a trend towards lower symptom scores,^26–29^ potentially explaining time trends in improved outcomes and lower mortality rates. Delivering reperfusion therapy to elderly patients appears to have been beneficial and may have contributed to the positive trend observed in outcomes within this age group. However, assessing causal inferences for these trends lies beyond this study’s scope, necessitating further investigations. The absence of outcome trends in the MT and IVT + MT groups may be related to the recent implementation of MT and a low case count.

## Strengths and limitations

This study utilised a nationwide registry known for its completeness, validity and reliability.^30,31^ Given the register’s size and quality, the results reliably reflect the clinical practice in treating and managing AIS-patients.

The generalisability of our results may be limited, as the study focused on first-ever AIS patients within Norway’s public healthcare system, a high-income country with robust welfare system that may lower barriers to hospital contact. Norway’s relatively small and homogenous population with little immigration could also impact the applicability of these findings to other populations. Register data shortcomings include missing values exceeding 5% for some variables, including follow-up mRS score (Supplemental Table 3). Patients in NSR lost to follow-up at 90-day tend to be slightly older, more likely to live in a nursing home and to have a prestroke mRS-score of 2-4.^32^ Additionally, mRS-score is assessed by 50 different hospitals and numerous healthcare workers, introducing some uncertainty to the variable. However, there is no reason to suspect substantial changes in loss to follow-up and mRS-assessment during the study period, thus the impact on our results is likely minimal. Furthermore, we did not have information on other factors that are essential for comprehensive stroke care, such as changes in stroke unit care, brain protection measures and improvements in rehabilitation therapies.

## Conclusion

The use of reperfusion therapies for first-ever AIS patients increased significantly over time, concurrent with significant improvements in functional outcome and lower mortality rates. These improvements over time were more prominent in older AIS population. Improved outcome in non-reperfused suggest other factors than reperfusion therapy could contribute to these results.

## Supplemental Material

sj-docx-1-eso-10.1177_23969873251331482 – Supplemental material for Trends in reperfusion treatments, functional outcomes and mortality for first-ever ischaemic stroke in Norway from 2014 to 2021: The Norwegian Stroke Registry Supplemental material, sj-docx-1-eso-10.1177_23969873251331482 for Trends in reperfusion treatments, functional outcomes and mortality for first-ever ischaemic stroke in Norway from 2014 to 2021: The Norwegian Stroke Registry by Kevin C Elangwe, Ellisiv B Mathiesen, Torunn Varmdal, Bent Indredavik and Agnethe Eltoft in European Stroke Journal
