# Supplementary material for: Trends in reperfusion treatments, functional outcomes and mortality for first-ever ischaemic stroke in Norway from 2014 to 2021: The Norwegian Stroke Registry
Source: Eur Stroke J. 2025 Apr 13;10(4):1445–53. doi: 10.1177/23969873251331482 (PMC11994638; doi:10.1177/23969873251331482)
Supplement: sj-docx-1-eso-10.1177_23969873251331482 – Supplemental material for Trends in reperfusion treatments, functional outcomes and mortality for first-ever ischaemic stroke in Norway from 2014 to 2021: The Norwegian Stroke Registry [file sj-docx-1-eso-10.1177_23969873251331482.docx]

**Supplemental material**

**Trends in functional outcomes and mortality stratified by treatment group for acute ischemic stroke in Norway from 2014 to 2021. The Norwegian Stroke Registry.**

Authors: Kevin C. Elangwe, Ellisiv B. Mathiesen, Torunn Varmdal, Bent Indredavik, Agnethe Eltoft

**Supplemental Table 1. Functional status, living conditions and mortality at follow-up, stratified by treatment modality**

|  | **2014** | **2015** | **2016** | **2017** | **2018** | **2019** | **2020** | **2021** | **p-value*** |
| --- | --- | --- | --- | --- | --- | --- | --- | --- | --- |
| **IVT** | | | | | | | | | |
| **Functional status at three months follow-up, n (%)** | | | | | | | | | |
| Excellent (mRS 0-1) | 287 (42.6) | 357 (44.5) | 377 (44.0) | 441 (44.5) | 461 (45.9) | 453 (43.8) | 528 (52.1) | 513 (51.2) | <0.0001 |
| Good (mRS 0-2) | 369 (54.8) | 466 (58.0) | 534 (62.3) | 601 (60.6) | 653 (65.0) | 647 (62.5) | 671 (66.2) | 686 (68.5) | <0.0001 |
| Moderate (mRS 3-4) | 124 (18.4) | 151 (18.4) | 132 (15.4) | 136 (13.7) | 120 (12.0) | 133 (12.9) | 143 (14.1) | 124 (12.4) | <0.0001 |
| Poor (mRS 5-6) | 110 (16.3) | 120 (14.9) | 113 (13.2) | 149 (15.0) | 120 (12.0) | 133 (12.9) | 116 (11.5) | 115 (11.5) | 0.0009 |
| **Discharge home, n (%)** | 371 (45.0) | 422 (47.7) | 477 (49.2) | 566 (51.5) | 578 (52.1) | 568 (51.5) | 599 (54.5) | 608 (55.8) | <0.0001 |
| **Mortality at different time points from symptom onset, n (%)** | | | | | | | | | |
| 30 days | 82 (10.0) | 83 (9.4) | 80 (8.3) | 108 (9.9) | 84 (7.6) | 79 (7.2) | 72 (6.6) | 72 (6.6) | 0.0001 |
| 90 days | 98 (11.9) | 106 (12.0) | 99 (10.2) | 138 (12.6) | 104 (9.4) | 109 (9.9) | 93 (8.5) | 98 (9.0) | 0.0006 |
| 1 year | 147 (17.8) | 140 (15.8) | 152 (15.7) | 196 (17.9) | 151 (13.6) | 161 (14.6) | 137 (12.5) | 143 (13.1) | <0.0001 |
| **MT** |  |  |  |  |  |  |  |  |  |
| **Functional status at three months follow-up, n (%)** | | | | | | | | | |
| Excellent (mRS 0-1) | 4 (20.0) | 6 (21.4) | 9 (22.5) | 13 (25.0) | 23 (22.8) | 20 (16.3) | 33 (24.3) | 26 (16.5) | 0.483 |
| Good (mRS 0-2) | 8 (40.0) | 10 (35.7) | 16 (40.0) | 22 (42.3) | 39 (38.6) | 34 (27.6) | 50 (36.8) | 51 (32.3) | 0.325 |
| Moderate (mRS 3-4) | 4 (20.0) | 8 (28.6) | 11 (27.5) | 11 (21.2) | 26 (25.7) | 32 (26.0) | 39 (28.7) | 53 (33.5) | 0.119 |
| Poor (mRS 5) | 5 (25.0) | 8 (28.6) | 10 (25.0) | 11 (21.2) | 29 (28.7) | 42 (34.1) | 38 (27.9) | 43 (27.2) | 0.991 |
| **Discharge: home, n (%)** | 6 (27.3) | 7 (23.3) | 5 (10.9) | 12 (21.8) | 29 (24.4) | 31 (17.8) | 31 (20.1) | 27 (15.9) | 0.334 |
| **Mortality at different time points from symptom onset, n (%)** | | | | | | | | | |
| 30 days | 3 (13.6) | 4 (13.3) | 8 (17.4) | 8 (14.8) | 18 (15.1) | 25 (14.4) | 30 (19.4) | 28 (16.5) | 0.635 |
| 90 days | 4 (18.2) | 6 (20.0) | 9 (19.6) | 9 (16.7) | 24 (20.2) | 37 (21.3) | 34 (21.9) | 36 (21.2) | 0.719 |
| 1 year | 7 (31.8) | 8 (26.7) | 13 (28.3) | 13 (24.1) | 31 (26.1) | 50 (28.7) | 39 (25.2) | 46 (27.1) | 0.509 |
| **IVT + MT** |  |  |  |  |  |  |  |  |  |
| **Functional status at three months follow-up, n (%)** | | | | | | | | | |
| Excellent (mRS 0-1) | 12 (30.0) | 16 (22.5) | 18 (21.2) | 47 (33.8) | 54 (30.7) | 36 (21.6) | 51 (28.3) | 61 (31.6) | 0.289 |
| Good (mRS 0-2) | 19 (47.5) | 30 (42.3) | 36 (42.4) | 71 (51.1) | 88 (50.0) | 70 (41.9) | 78 (43.3) | 109 (56.5) | 0.084 |
| Moderate (mRS 3-4) | 12 (30.0) | 21 (29.6) | 24 (28.2) | 36 (25.9) | 36 (20.5) | 37 (22.2) | 43 (23.9) | 39 (20.2) | 0.038 |
| Poor (mRS 5) | 5 (12.5) | 13 (18.3) | 18 (21.2) | 27 (19.4) | 34 (19.3) | 43 (25.7) | 49 (27.2) | 34 (17.6) | 0.417 |
| **Discharge: home, n (%)** | 9 (19.1) | 19 (25.7) | 27 (27.6) | 38 (24.1) | 38 (20.2) | 49 (22.2) | 50 (27.0) | 58 (28.4) | 0.186 |
| **Mortality at different time points from symptom onset, n (%)** | | | | | | | | | |
| 30 days | 3 (6.4) | 10 (13.5) | 12 (12.4) | 20 (12.6) | 25 (13.3) | 30 (13.6) | 30 (16.1) | 20 (9.9) | 0.948 |
| 90 days | 5 (10.6) | 10 (13.5) | 16 (16.5) | 23 (14.5) | 30 (16.0) | 35 (15.8) | 35 (18.8) | 26 (12.8) | 0.962 |
| 1 year | 5 (10.6) | 13 (17.6) | 17 (17.5) | 28 (17.6) | 37 (19.7) | 45 (20.4) | 47 (25.3) | 36 (17.7) | 0.288 |
| **No IVT/MT** |  |  |  |  |  |  |  |  |  |
| **Functional status at three months follow-up, n (%)** | | | | | | | | | |
| Excellent (mRS 0-1) | 1603 (43.6) | 1535 (39.1) | 1548 (40.7) | 1592 (41.4) | 1622 (41.2) | 1643 (40.4) | 1834 (45.0) | 1820 (44.1) | 0.0005 |
| Good (mRS 0-2) | 2081 (56.6) | 2207 (56.2) | 2185 (57.5) | 2208 (57.5) | 2322 (59.0) | 2418 (59.5) | 2518 (61.8) | 2505 (60.7) | <0.0001 |
| Moderate (mRS 3-4) | 543 (14.8) | 611 (15.6) | 592 (15.6) | 600 (15.6) | 575 (14.6) | 584 (14.4) | 658 (16.1) | 615 (14.9) | 0.892 |
| Poor (mRS 5) | 574 (15.6) | 598 (15.2) | 534 (14.1) | 567 (14.8) | 549 (13.9) | 557 (13.7) | 546 (13.4) | 543 (13.2) | 0.0009 |
| **Discharge: home, n (%)** | 2299 (51.8) | 2272 (51.3) | 2291 (51.7) | 2191 (50.4) | 2276 (52.6) | 2309 (52.6) | 2456 (55.1) | 2542 (56.0) | <0.0001 |
| **Mortality at different time points from symptom onset, n (%)** | | | | | | | | | |
| 30 days | 359 (8.1) | 378 (8.5) | 342 (7.7) | 355 (8.2) | 314 (7.3) | 299 (6.8) | 331 (7.4) | 325 (7.2) | 0.0081 |
| 90 days | 516 (11.6) | 530 (11.9) | 462 (10.4) | 489 (11.3) | 463 (10.7) | 445 (10.1) | 449 (10.1) | 465 (10.2) | 0.0024 |
| 1 year | 794 (17.9) | 787 (17.7) | 732 (16.5) | 780 (18.0) | 754 (17.4) | 713 (16.2) | 689 (15.5) | 699 (15.4) | <0.0001 |

IVT: intravenous thrombolysis, MT: mechanical thrombectomy, mRS: modified Rankin Scale

*p for time trend, assessed by logistic regression adjusted for age and sex

**Supplemental Table 2. P-values for interaction with time by age, sex, hospital size and NIHSS for reperfusion treatment and outcome**

|  | **Age**  < 80 vs. ≥ 80 | **Sex**  men vs. women | **Hospital size**  0-99, 100-299 vs. ≥ 300 | **NIHSS**  < 5 vs. ≥ 5 |
| --- | --- | --- | --- | --- |
| IVT | 0.980 | 0.571 | 0.6697 | **0.0003** |
| MT | **0.0041** | 0.611 | **0.0005** | 0.615 |
| IVT+MT | 0.172 | 0.861 | **0.0023** | 0.670 |
| **mRS three-months** | | | | |
| Excellent (0-1) | **<0.0001** | 0.121 | 0.361 | 0.577 |
| Good (0-2) | **0.0003** | **0.0143** | 0.491 | 0.147 |
| Moderate (3-4) | 0.666 | 0.312 | 0.499 | 0.129 |
| Poor (5-6) | **0.0013** | 0.1126 | 0.1293 | 0.946 |
| Discharge: Home | **0.0044** | 0.1469 | 0.07034 | 0.5504 |
| **Mortality** | | | | |
| 30-days | **0.0018** | 0.3662 | 0.489 | 0.204 |
| 90-days | **0.0002** | 0.1642 | 0.270 | 0.2479 |
| 1-year | **<0.0001** | **0.0203** | 0.750 | 0.188 |
| NIHSS: National Institutes of Health Stroke Scale.  Interaction in time trends by age, sex, baseline NIHSS-score and hospital size were assessed by including cross-products of these variables with time in age and sex adjusted binary logistic regression models for reperfusion treatment and outcome trends. P values < 0.05 are presented in bold. | | | | |


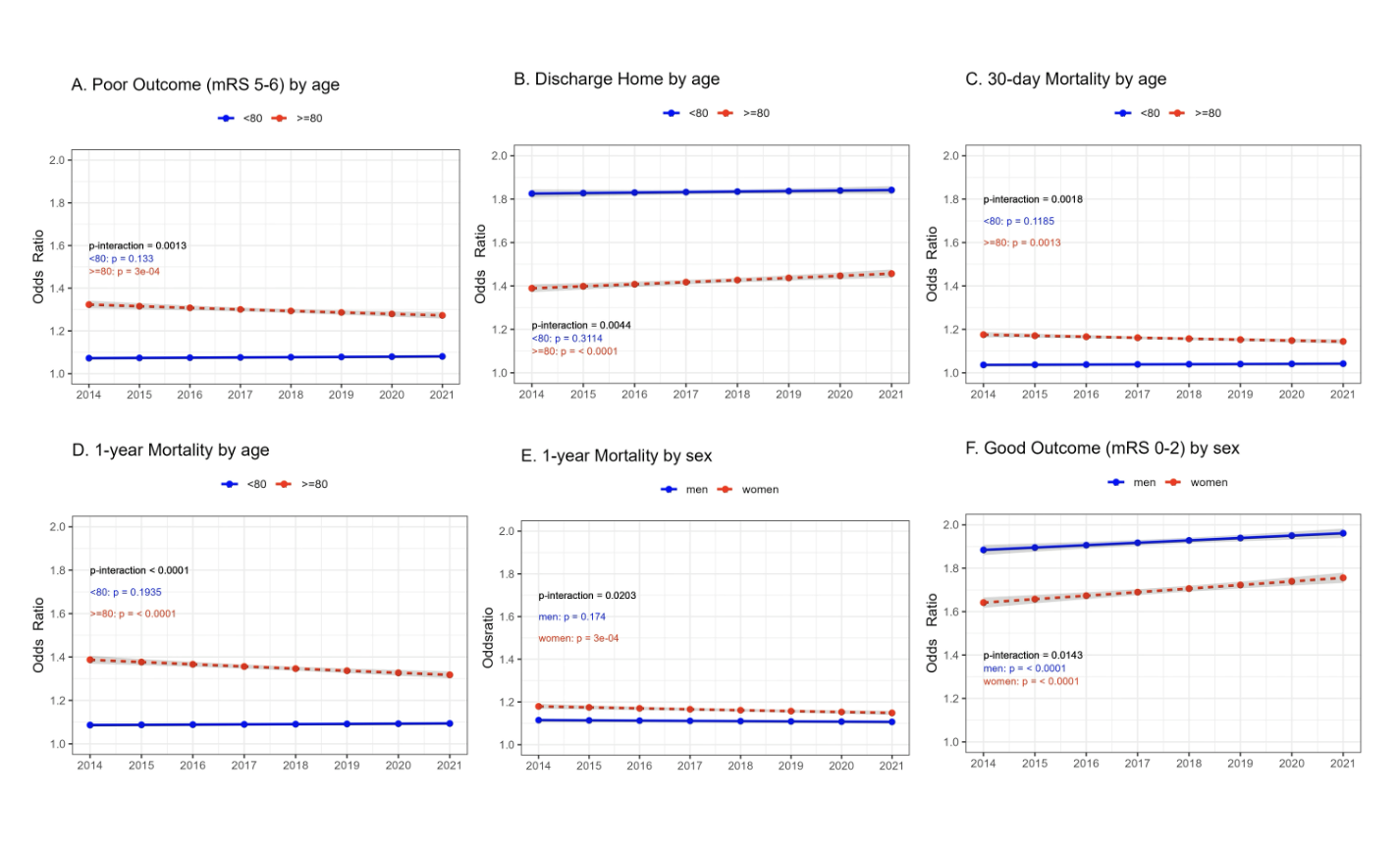
 **Supplemental figure 1**: Time trends in odds ratio for different outcomes after first-ever acute ischemic stroke stratified by age and sex. Significant interactions not presented in main manuscript are displayed here.


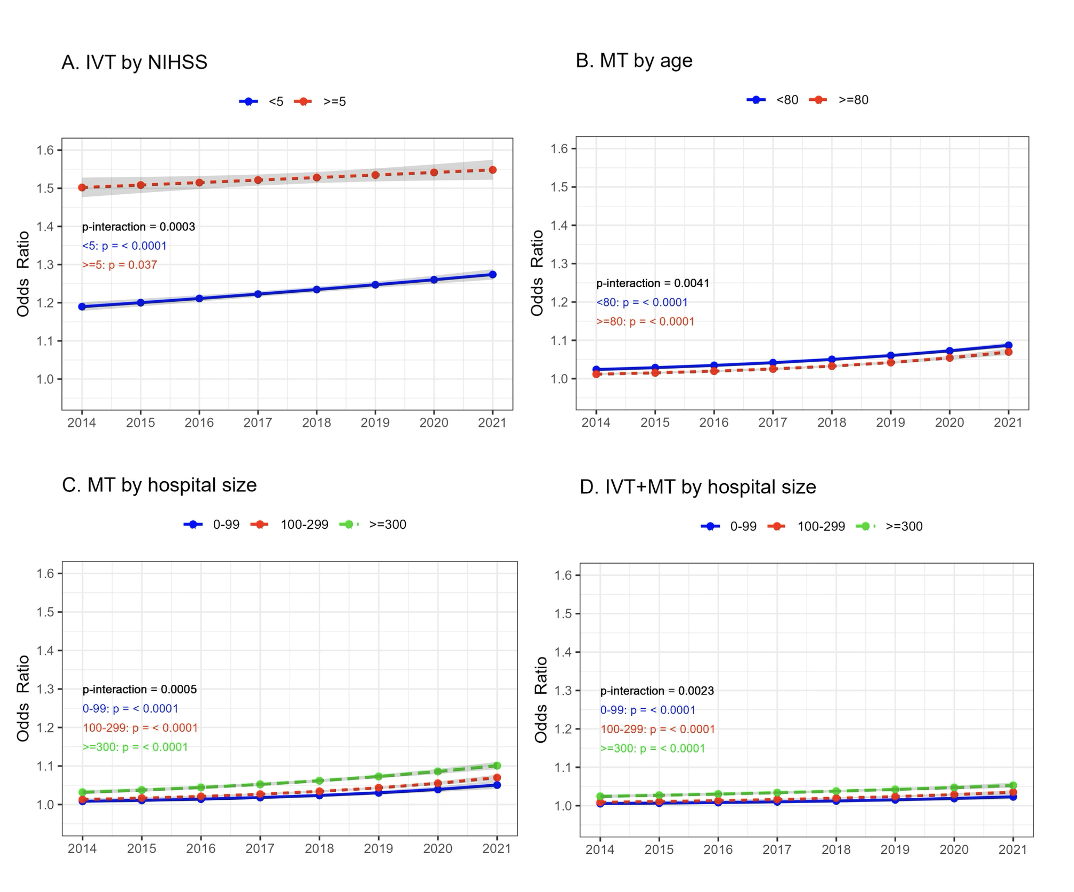


**Supplemental figure 2**: Time trends in odds ratio for reperfusion treatment after first-ever acute ischemic stroke stratified by stroke severity, age and hospital size. Significant interactions not presented in main manuscript are displayed here. IVT: intravenous thrombolysis, MT: mechanical thrombectomy, NIHSS: National Institutes of Health Stroke Scale

**Supplemental Table 3. Missing data, n = 45 686**

| Variable | Missing, n | Missing, % |
| --- | --- | --- |
| Age | 0 | 0 |
| Sex | 0 | 0 |
| Living arrangement | 228 | <0.01 |
| Smoking status | **7133** | **15.6** |
| Prior TIA | 428 | <0.01 |
| Prior myocardial infarction | 263 | <0.01 |
| Diabetes | 174 | <0.01 |
| Atrial Fibrillation | 304 | <0.01 |
| Antihypertensive drugs | 365 | <0.01 |
| Cholesterol-lowering drugs | 292 | <0.01 |
| Oral anticoagulants | 247 | <0.01 |
| Platelets inhibitors | 235 | <0.01 |
| Pre-stroke mRS | 3212 | 0.07 |
| NIHSS on admission | **8989** | **19.7** |
| IVT | 69 | <0.01 |
| MT | 99 | <0.01 |
| Stroke unit | 0 | 0 |
| mRS follow-up | **9643** | **21.1** |
| Discharge | 41 | <0.01 |
| Mortality | 35 | <0.01 |

IVT: intravenous thrombolysis, MT: mechanical thrombectomy, NIHSS: National Institutes of Health Stroke Scale.

Variables with missing values >5% are displayed in bold.
